# Disambiguating ambiguous motion perception: what are the cues?

**DOI:** 10.3389/fpsyg.2015.00902

**Published:** 2015-07-09

**Authors:** Alessandro Piedimonte, Adam J. Woods, Anjan Chatterjee

**Affiliations:** ^1^Department of Psychology, University of TurinTurin, Italy; ^2^Department of Neurology, Center for Cognitive Neuroscience, University of PennsylvaniaPhiladelphia, PA, USA; ^3^Center for Cognitive Aging and Memory, Institute on Aging, Department of Aging and Geriatric Research, University of FloridaGainesville, FL, USA

**Keywords:** motion perception, associative learning, ambiguous motion, top–down, bottom–up

## Abstract

Motion perception is a fundamental feature of the human visual system. As part of our daily life we often have to determine the direction of motion, even in ambiguous (AMB) situations. These situations force us to rely on exogenous cues, such as other environmental motion, and endogenous cues, such as our own actions, or previously learned experiences. In three experiments, we asked participants to report the direction of an AMB motion display, while manipulating exogenous and endogenous sources of information. Specifically, in all three experiments the exogenous information was represented by another motion cue while the endogenous cue was represented, respectively, by movement execution, movement planning, or a learned association about the motion display. Participants were consistently biased by less AMB motion cues in the environment when reporting the AMB target direction. In the absence of less AMB exogenous motion information, participants were biased by their motor movements and even the planning of such movements. However, when participants learned a specific association about the target motion, this acquired endogenous knowledge countered exogenous motion cues in biasing participants’ perception. Taken together, our findings demonstrate that we disambiguate AMB motion using different sources of exogenous and endogenous cues, and that learned associations may be particularly salient in countering the effects of environmental cues.

## Introduction

The ability to detect a movement in the environment is critical to survival. It is maintained across different species, and is equally important for predator and prey (Rokszin et al., 2010). Tracking the direction of movements in the visual environment is often easy but motion perception can also be hampered by ambiguity in some situations. For example, predicting turns of other cars on the road may be difficult on a foggy day. However, multiple sources of information, exogenous, or endogenous, can help to solve this visual ambiguity.

The effects of exogenous and endogenous cues have been studied for over a century, especially in the context of spatial attention (e.g., James, 1890; Corbetta and Shulman, 2002). Classically, attention driven by external events is referred to as bottom–up or exogenous attention whereas goal-driven attention is referred as top–down or endogenous attention (Posner and Cohen, 1984; MacLean et al., 2009). For instance, while driving, we could look at exogenous cues such as direction indicators and predict which direction cars might go (i.e., an exogenous source of information). Features, like colors, shapes and motion, are automatically extracted from the visual environment by our brain in *bottom–up* processes (Folk et al., 1994). This automatic feature extraction applies also to complex stimuli. For instance, even if we do not know where a specific bird in a flock is flying, we can predict its path because our brain automatically extracts the flock’s direction. This phenomenon, called motion capture, links different features of complex environmental stimuli.

On the other hand, we can use prior knowledge to select specific sensory information. This *top–down* process helps to disambiguate ambiguous (AMB) bottom–up information. For instance, on certain roads we might know that cars move from left to right and; hopefully, not in the opposite direction or we could use cues from our past experience on the same road and we might remember that the road turns right, so that other cars will likely go in this direction (i.e., endogenous sources of information). Other top–down processes, such as expectations, can bias also our perception. For instance, when an occluded horizontal row of shapes is shifted laterally, apparent motion can he experienced in either the leftward or the rightward direction but observers looking at a certain shape (e.g., a triangle facing left) experience movement in the direction that the shape appears to face (i.e., left). The similar effect also occurs with biological figures (e.g., a mouse) moving ambiguously: in this case people experience movement in the direction of the biologically expected movement (e.g., in front of the mouse); the forward-facing attribute of an AMB shape biases its perceived direction (McBeath et al., 1992; McBeath and Morikawa, 1997).

Voluntary actions can also influence motion perception (Ishimura and Shimojo, 1994; Wohlschläger, 2000). Even though neuroscience has investigated the link between perception and action for the last 30 years, these studies typically focus on the influence of visual cues on movement (e.g., Lee, 1999; for a review, see Goodale, 2011), rather than the influence of actions on visual perception (e.g., Wohlschläger, 2000).

Research investigating the influence of actions on AMB visual motion have used apparent motion displays. When presented with a sequence of static images showing different positions of a moving object, we perceive the object moving fluidly (Wertheimer, 1912). Television and movies capitalize on this “apparent motion” phenomenon, which has been well-studied in visual neuroscience (Kolers, 1972; Ramachandran and Anstis, 1983). Brain areas active during actual motion perception, V1 and middle-temporal area (MT), are also active during apparent motion perception (Williams et al., 2003; Muckli et al., 2005). It is also possible to create ambiguity in such stimuli so that the perceived direction is unpredictable and may reverse after few seconds analogs to how depth can reverse with the Necker cube (Ilg et al., 2008). Such AMB motion can be biased by hand movements. If participants move their hands they perceive the AMB motion as moving in the same direction as their hand, even when their hand is not visible (Ishimura, 1995; Wohlschläger, 2000).

However, it is still not clear whether our actions bias our perception (i.e., endogenous cues) when we are faced with exogenous cues from the environment – a typical situation encountered frequently. When we move in a certain direction but are exposed to an opposing exogenous cue (i.e., an opposite direction), will our perception remain biased by our movement? Is there a difference between action and the plan to act? In the previous example of driving a car, it might be too late to recognize that a sign indicates a dead end since we are already moving in the wrong direction (i.e., we are already in the middle of performing an action). Also, what is the role of our past experience in this process? In a previous study, Haijiang et al. (2006) showed that associating a strong directional signal to a noisy target (a bistable rotating Necker cube) through classical conditioning changes the target’s perceived direction so that it is perceived as rotating in the same direction as the signal. Also, using a different paradigm involving an AMB motion display (composed by randomly moving dots), it has been shown that observers can be induced to organize the AMB motion and report its speed by using weak embedded motion signals (Durgin, 2002).

In the present research, we sought to extend our understanding of the processes that resolve perceptions of AMB motion, examining how these different sources of information, represented by exogenous and endogenous cues, can bias our perception of motion when the motion itself is inherently AMB.

Specifically, the first experiment tested the hypothesis that voluntary movements bias our motion perception, regardless of the direction of exogenous cues. The second experiment tested the hypothesis that a planned, but not yet performed, action would have similar effects as an explicitly executed action. Finally, we tested the hypothesis that prior knowledge biases our perception of AMB motion. Results from the three experiments provide new insight into (1) perception of AMB motion, (2) the sources of information that bias our perception and, (3) the relationship between top–down and bottom–up processes in disambiguating motion.

## Materials and Methods

### Participants

Sixteen college age participants were recruited from the University of Pennsylvania for each experiment (total *n* = 48, see **Table 1** for demographics). Handedness was assessed using a modified version of the Annett’s (1967) questionnaire (score range = from -24, completely left-handed, to +24, completely right-handed; Briggs and Nebes, 1975). Left-handed participants (i.e., with a handedness score between -8 and -24) and participants with vision problems were excluded from participation. All participants gave written informed consent prior to participation in the study. The University of Pennsylvania’s Institutional Review Board approved the study.

**Table 1:** Demographic Information.

	Age and gender	Education	Handedness
Experiment 1 (*n* = 16)	23.4 ± 3.3 years; 9 F	16.3 ± 2 years	19 ± 5.7
Experiment 2 (*n* = 16)	19.4 ± 1.5 years; 10 F	14.6 ± 1.3 years	17.8 ± 5.5
Experiment 3 (*n* = 16)	20.5 ± 3 years; 10 F	14.5 ± 1.6 years	17.5 ± 6.9

*Mean ± SD is shown for age, education, and handedness*.

### Stimuli and Apparatus Common to All Experiments

The stimuli used in this study were created with a free vector graphics editor (Inkscape, ver 0.48.2, The Inkscape Team). The programming scripts were created and executed using Presentation (ver 14.6; Neurobehavioral Systems, Inc.) on a PC laptop (HP TouchSmart tx2 1050 el). The stimuli were displayed on a monitor (height = 30 cm; length = 39 cm) linked to a laptop and positioned on a table. Participants sat approximately 71 cm in front of the monitor with their trunk midline aligned to the monitor midline. An experimenter was always present in the room where participants were performing the experiments and controlled that participants position on the chair to ensure that the viewing distance and trunk position did not change during the experiments.

**FIGURE 1:**
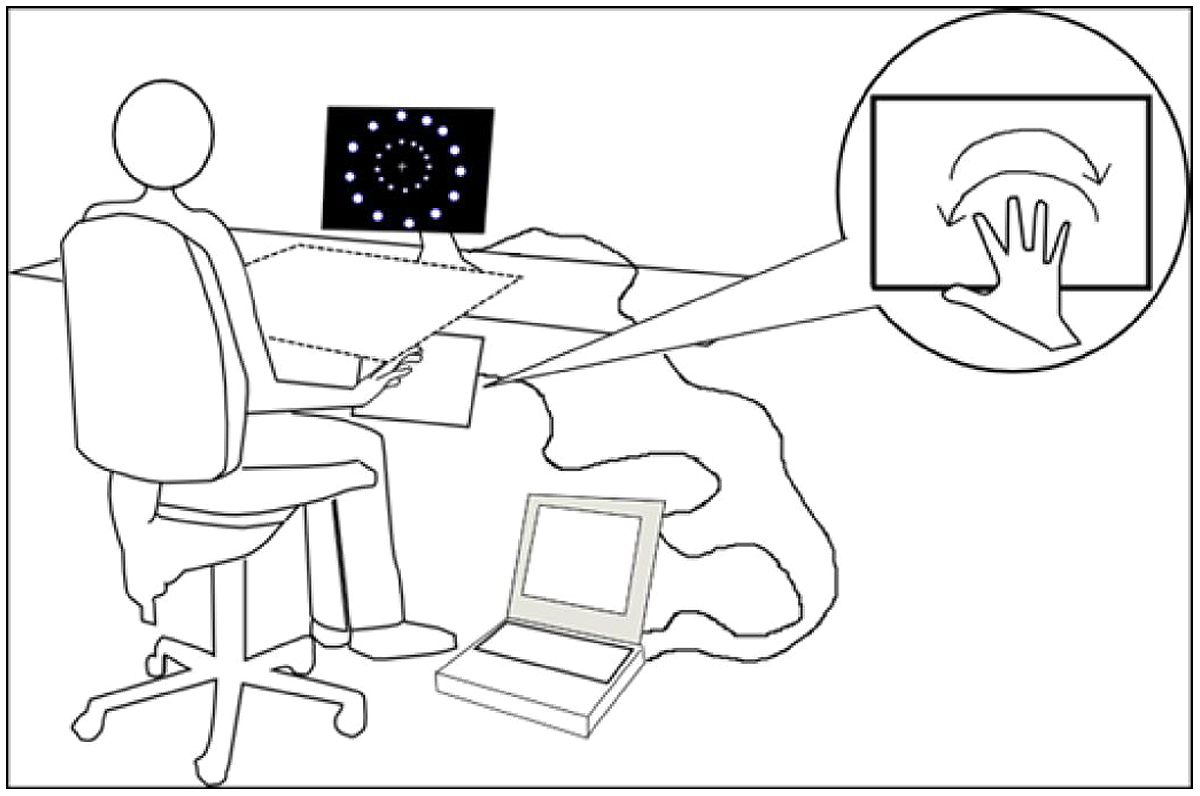
**Participant position, apparatus, and stimuli representation**.

In the first and second experiment, a touch-screen (Magic Touch; Keytec, Inc.) was linked to the laptop and positioned at a comfortable distance based on the reach of each participant. The touch-screen and the participants’ arms were covered by a square box (65 cm × 65 cm). In the first two experiments, the experimenter present in the room made sure that all participants performed the correct movements required. In the third experiment the box and the touch-screen were removed (see **Figure 1**) since participants did not perform or plan arms movements (see Experiment 3).

**FIGURE 2:**
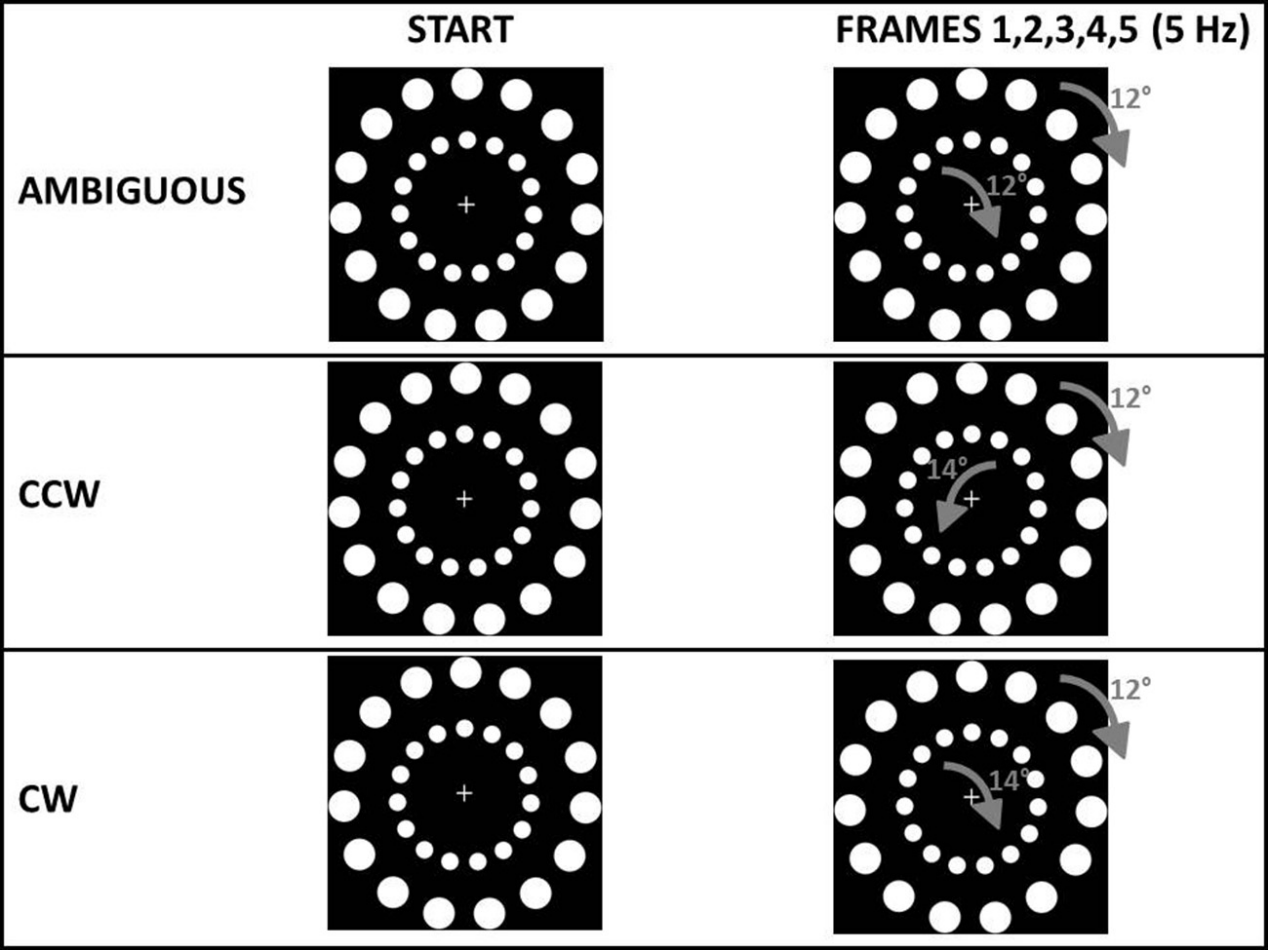
**Type of stimuli common to all experiments.** In the ambiguous (AMB) condition both the outer circle and the inner circle were shifted by 12° to the right. In the counterclockwise condition (CCW) the outer circle was shifted by 12° to the right while the inner circle was shifted by 14° to the left. In the clockwise condition (CW) the outer circle was shifted by 12° to the right while the inner circle was shifted by 14° to the right. In all experiments both the outer and the inner circle were shifted five times per second (5 Hz). Participants were requested to judge only the rotation of the outer circle.

For all experiments, the main stimuli consisted of two circles one nested within the other (see **Figure 2**). Stimuli were based on a stimulus created by Ilg et al. (2008). Each circle contained 15 white dots on a black background (24° between each other). The outer circle had a diameter of 19.3 cm and contained dots with a diameter of 2.2 cm. The inner circle had a diameter of 10.3 cm and contained dots with a diameter of 1.2 cm. A fixation cross was displayed in the middle of the stimulus (see **Figure 2**). During each trial of every experiment, different frames of the circles were displayed at a frequency of 5 Hz, rotated by a specific angle: this shift between frames gave the participant the perception of a continuous rotation. The outer circle always rotated by +12° in each frame. In a pilot study (*n* = 12) we asked participants, in 30 trials, to tell their perceived direction of a circle with the same structure of the outer circle in our experiments (15 dots, 24° between each other, shifted by +12° at 5 Hz). In this pilot study we confirmed that a +12° shift resulted in an AMB (either clockwise, CW or counterclockwise, CCW) perception of motion (mean CW perceptions = 16.08) confirming early results with this type of stimulus (Ilg et al., 2008). In all the experiments, the main request to the participants was: “in which direction do you perceive the outer circle to be rotating?” The movement of the inner circle represented exogenous information from the environment. The circular motion was selected as the exogenous cue for two reasons. First, motion is a salient visual characteristic, perhaps more so than color, intensity, and orientation (Itti, 2005). Second, since the exogenous cue and the stimulus to be judged were similar (see **Figure 2**) we expected participants to easily group them together, following the classic Gestalt rule of similarity (Torodovic, 2008) even when instructed to ignore the inner circle. Changing the angular displacement of the inner circle’s dots created three perceived rotations: an AMB rotation (equal to the outer circle’s rotation), a CW rotation and a CCW rotation. These three inner circle rotations were used to bias the perception of the outer circle’s rotation. The inner circle’s rotation was AMB in one third of the trials using the same shifting angle and frequency as the outer circle’s rotation (i.e., shifted by 12° to the right in each frame at 5 Hz). To choose an angle that could create a rotation perceived as CW, in a second pilot study (*n* = 12) we asked participants to tell their perceived direction of a circle with the same structure of the outer circle in our experiments (15 dots, 24° between each other) shifted by different angles (+12°, +14°, -14°) at 5 Hz, with 20 trials for each angle (total trials = 60). In this second pilot study we first confirmed that a +12° results in an AMB bias (mean CW perceptions = 10.57) while we found that rotating the 15 dots circle by 14° to the right with the same frequency of 5 Hz, created a strong CW bias (mean CW perceptions ± SE = 17.42) and the opposite rotation, that is 14° to the left, created a strong CCW bias (mean CW perceptions = 2.42). Using these two angles (+14° and -14°): in other third of the trials the inner circle’s rotation was rendered to be perceived as CW (angle = +14°, freq = 5 Hz) and in the last third of the trials perceived as CCW (angle = -14°, freq = 5 Hz).

Thus, in each experiment, participants received three different types of stimuli (see **Figure 2**):

– An outer circle and an inner circle both perceivable as rotating ambiguously (both shifted by 12°; AMB stimulus).
– An outer circle perceivable as rotating ambiguously (12°) while the inner circle was more readily perceived as rotating CW (+14°; CW stimulus).
– An outer circle perceivable as rotating ambiguously (12°) while the inner circle was more readily perceived as rotating CCW (-14°; CCW stimulus).

Participants reported the direction of the outer circle’s rotation, which was always AMB, by saying “left” (CCW rotation perceived) or “right” (CW rotation perceived). In all experiments, participants reported their first perceptually unambiguous directional percept. Following this report, a black screen appeared and after 2 s another trial began.

In the first stage of training, participants were familiarized with the stimuli by judging the rotation of the outer circle in six trials (two trials for each stimulus type: AMB, CW, and CCW). This training was conducted in all three experiments.

## Experiment 1

### Procedure and Conditions

**FIGURE 3:**
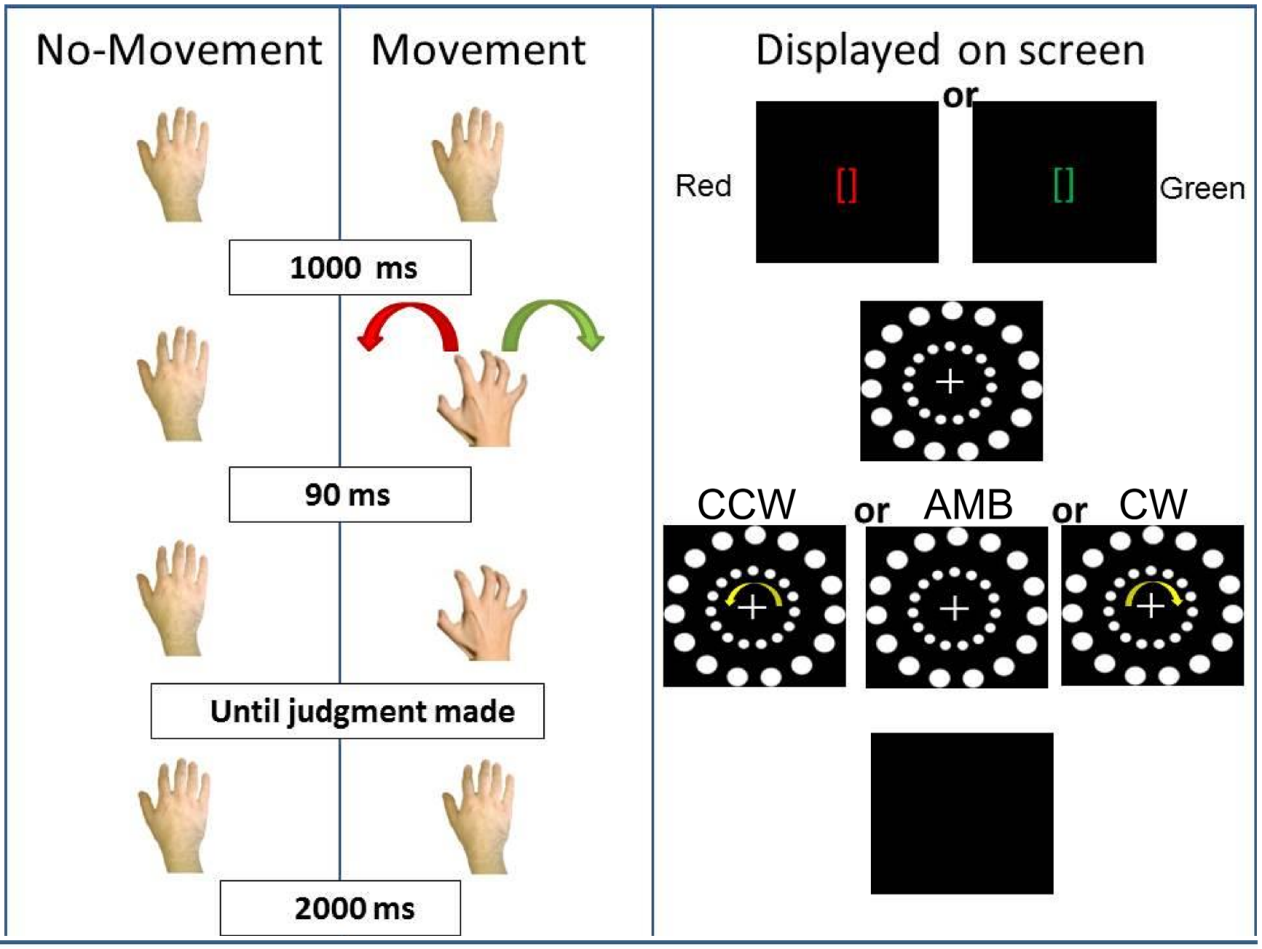
**Experiment 1 conditions.** On the **(Left)**, schematic representation of the two conditions: No-Movement and Movement. On the **(Right)**, the display on the screen during the trials. CCW = counterclockwise (inner circle shift = -14°), AMB = Ambiguous (inner circle shift = +12°), CW = clockwise (inner circle shift = +14°). Note that the outer circle is always shifted by +12°.

We tested the hypothesis that voluntary movements in a certain direction bias participants’ perception of an AMB visual display regardless of other visual cues. To test this hypothesis, we asked participants to perform movements with their hands (endogenous information) and then judge the direction of movement of an outer circle while the inner circular motion (exogenous information) was also displayed (see **Figure 3**). The first experiment, as well as the next one (Experiment 2), was motivated by the seminal paper written by Wohlschläger (2000) on visual motion priming.

In the first (No-Movement) condition, participants first fixate on green or red brackets ([]) in the middle of the screen (duration = 1000 ms). After the brackets disappeared, the stimulus composed of the two circles with a central cross appeared in the middle of the screen and subjects were instructed to fixate on the cross. After 90 ms, both circles started moving and participants were asked to report the direction of the rotation of the outer circle. The researcher present in the room recorded the judgment. Then, the screen turned black for 2000 ms before the start of the next trial. In this condition, we predicted that participants’ perception of the outer circle movement direction would be captured by the inner circle rotation. Participants would report more CW answers when the inner circle was perceived as rotating CW and more CCW answers when the inner circle was perceived as rotating CCW. When both circles were perceived as rotating ambiguously, we predicted that participants, at the group level, would not show a directional bias. This pattern of results would support the hypothesis that exogenous sources of information bias perception of AMB motion in the environment.

In the second (Movement) condition, the circles’ rotation was triggered by participants’ right hand movement. Participants first received two instructions (duration = 1000 ms): green brackets indicating a rightward (CW) hand rotation or red brackets indicating a leftward (CCW) hand rotation. After the brackets disappeared, the circles appeared and participants approached the touch-screen with their right hand and performed the requested movement. Ninety milliseconds after the start of the hand rotation, the circles started rotating and subjects were asked to report the direction of the outer circle’s rotation. The timing between hand rotation and circle rotation was decided because previous experiments have shown that an higher lag (e.g., 200 ms) can “separate” hand movement from its effect on visual perception (Hu and Knill, 2010). After the researcher recorded the judgment, the screen turned black for 2000 ms, and another trial started. In this condition, we predicted that actions would bias participants’ perception and would interact with the inner circle’s movements (exogenous information). If the inner circle was perceived as rotating ambiguously, participants’ hand movements would bias their perception. They would perceive more CW rotations when moving their hand CW and more CCW rotations when moving their hand CCW. If the inner circle was perceived as rotating in a certain direction (CW or CCW), we expected participants hand movements to have less influence when the hand was rotating in an opposite direction (interference) and greater influence when the hand and the inner circle were rotating in the same direction (facilitation).

For each cue (green or red brackets), 20 trials of each type of stimuli (AMB, CW, and CCW) were presented in each condition (120 trials in the No-Movement condition and 120 in the Movement condition) for a total of 240 trials per participant. Type of stimuli was randomized in each condition and conditions were counterbalanced within subjects in the following order: No-Movement, Movement, Movement, No-Movement. The experimenter present in the room registered verbal reports and made sure that all participants performed the correct movements required in the Movement condition. If a participant performed a wrong movement during a trial, the trial was repeated.

### Analysis and Results

**FIGURE 4:**
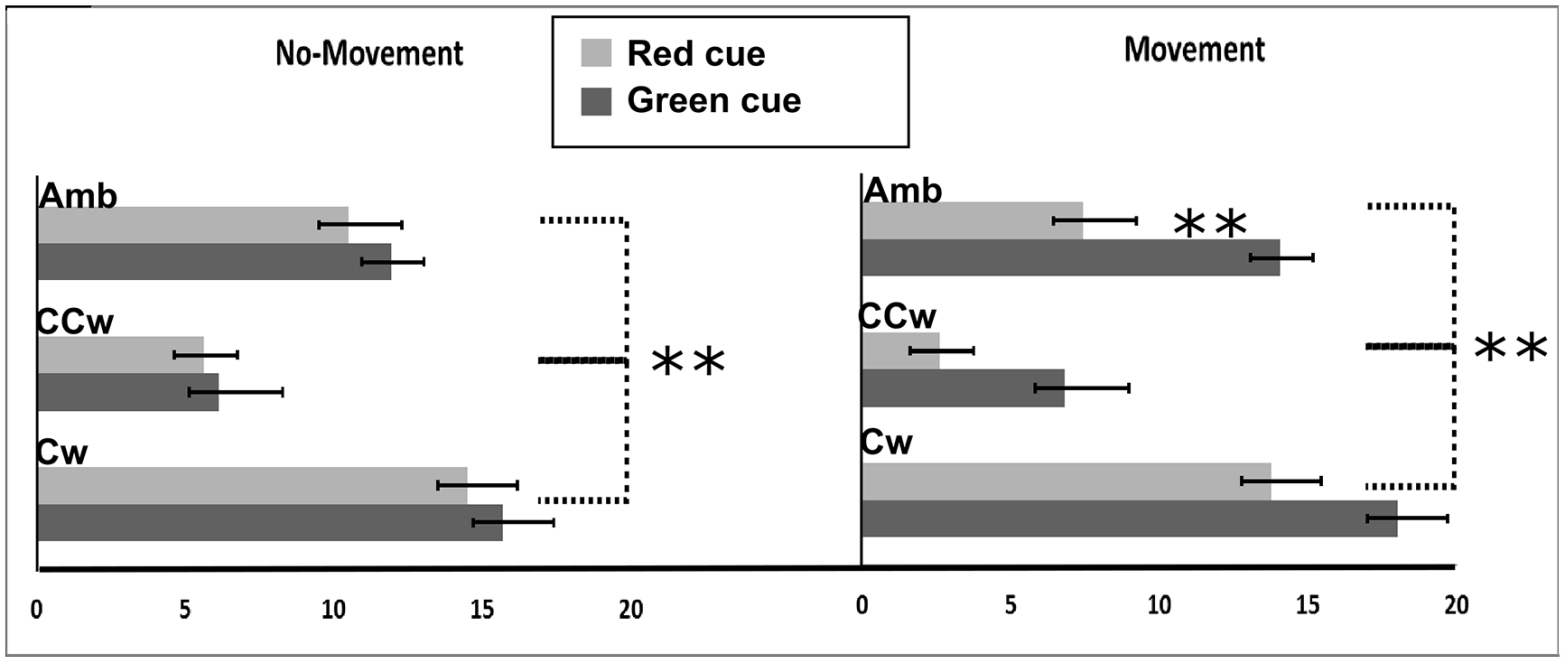
**Experiment 1 results.** On the *X***-**axis, perception index (PI) is shown (range: 0, +20). 0 represents a totally CCW biased perception while +20 represents a totally CW biased perception. On the *Y***-**axis Inner circle rotations are described: Amb = ambiguous inner circle rotation; CCw = counterclockwise circle rotation; Cw = clockwise inner circle rotation. Error bars represent SEM. Light gray bars represent red cues and dark gray bars represent green cues. Asterisks represent significant results: ^∗∗^*P* < 0.01.

We first calculated a Perception Index (PI) to represent the number of rightward (CW) directions of the outer circle perceived by each participant. The PI ranged between 0 (i.e., all CCW directions perceived) and +20 (i.e., all CW directions perceived). If the PI was 10 it indicated that that CW or CCW directions were equally perceived, while values lower than 10 indicated more CCW directions perceived and values higher than 10 indicated more CW directions perceived. All main results are illustrated in **Figure 4**. In particular, on the *Y*-axis, inner circle rotations are described (Amb = ambiguous inner circle rotation, +12°; CCw = counterclockwise inner circle rotation, -14°; Cw = clockwise inner circle rotation, +14°) while on the *X*-axis the value of PI is showed (between 0 and 20). Light gray bars represent red cues and dark gray bars represent green cues while error bars represent SEM. Asterisks represent significant results (^∗∗^*P* < 0.01). On the left part of the **Figure 4**, results from the No-Planning condition are showed while in the right part of the **Figure 4** results from the Planning condition are showed.

Using the PI as the dependent variable, we performed a 2 × 3 × 2 repeated measure ANOVA with three main factors: Condition (two levels, No-Movement or Movement), Inner rotation (three levels: AMB, CCW, CW), and Cue (two levels: red, green). The analysis showed a significant main effect of Inner Rotation [*F*(2,30) = 122.65; *P* < 0.001, $\eta_{p}^{2}$ = 0.89] and Cue [*F*(1,15) = 38.64; *P* < 0.001, $\eta_{p}^{2}$ = 0.72] and a significant interaction between Condition and Cue [*F*(1,15) = 12.58; *P* = 0.003, $\eta_{p}^{2}$ = 0.46]. Using Newman–Keuls *post hoc* tests to explore significant results, we found that Inner Rotation influence was explained by a difference between all three rotations: AMB, CCW, and CW (for each comparison: *P* < 0.001). Specifically, in both conditions (No-Movement and Movement), when the inner circle was perceived as rotating ambiguously participants did not have a specific bias in judging the outer circle rotation (mean = 10.984). When the inner circle was perceived as rotating CCW, participants were biased to judge the outer circle as rotating CCW (mean = 5.269). Finally, when the inner circle was perceived as rotating CW, participants were biased to judge the outer circle as rotating CW (mean = 15.484).

In the No-Movement condition, no significant difference was found between the green and the red cue, meaning that these cues did not bias their perception of the outer circle rotation. However, in the Movement condition, we found a significant difference in the perceived rotations based on the movement direction (*P <* 0.001^∗^). Participants perceived more CCW rotations when they moved CCW (mean = 7.93) and more CW rotations when they moved CW (mean = 12.95).

Furthermore, we conducted planned comparisons to check for interference or facilitation effects. Planned comparisons did not show interference effects between movements and inner circle rotations. Specifically, when participants moved their hand in an opposite direction with respect to the inner circle motion only the inner circle biased participants’ perceptions. However, planned comparisons showed significant facilitation effects between movements and inner circle rotations. Specifically, participants saw more CCW outer circle rotations when they moved their hand CCW and the inner circle was perceivable as rotating CCW than when they only saw the inner circle rotating CCW [*F*(1,15) = 6.13; *P* = 0.02]. Similar effects were seen with CW movements [*F*(1,15) = 5.45; *P* = 0.03].

### Discussion

Experiment 1 demonstrated that when the inner circle was perceived as rotating in a specific direction, participants judged the AMB rotation of outer circle as moving in the same direction. This result confirms that an exogenous source of movement bias our motion perception of AMB stimuli as also reported in other studies (Ball and Sekuler, 1981; Linares et al., 2012).

Secondly, we found that when participants moved (i.e., rotated) their hand in a specific direction and both circles were perceivable as rotating ambiguously, participants’ perception were biased toward the performed movement direction. This result shows that an endogenous source of information, here represented by a specific movement, bias motion perception of AMB stimuli, also confirming early studies (Wohlschläger, 2000).

When the endogenous and the exogenous sources of information were in opposition, only the exogenous (inner circular rotation) biased participants’ motion perception. When participants rotated their hand in the same direction as the inner circle, this shared direction biased their perception more so than when they just had access only to the exogenous information. This last finding indicates that, even though movement execution did not interfere with the bias from the inner circle rotation, it facilitated those effects on participants’ perception.

## Experiment 2

### Procedure and Conditions

**FIGURE 5:**
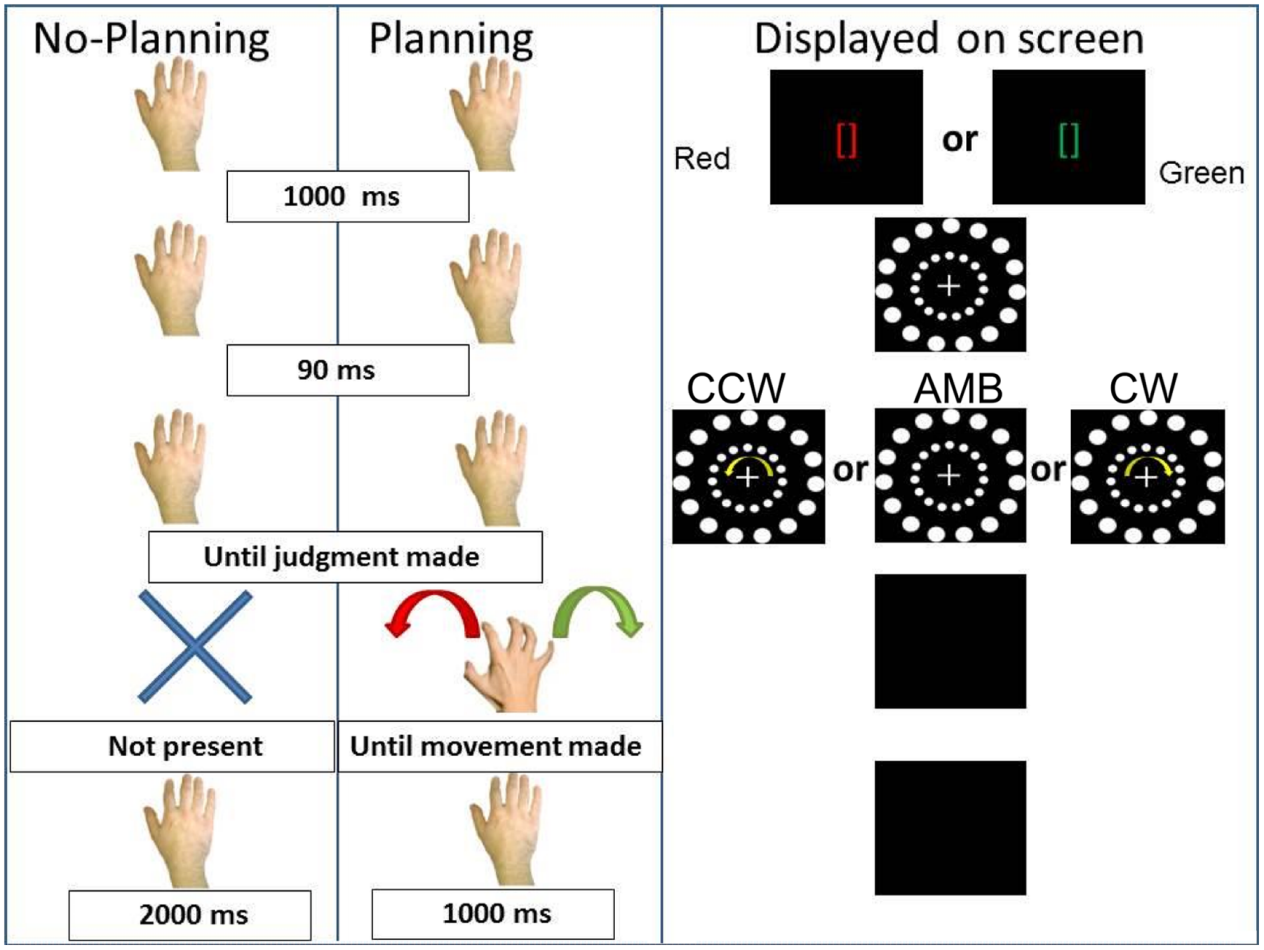
**Experiment 2 conditions.** On the **(Left)**, schematic representation of the two conditions: No-Planning and Planning. On the **(Right)**, the display on the screen during the trials. CCW = counterclockwise (inner circle shift = -14°), AMB = Ambiguous (inner circle shift = +12°), CW = clockwise (inner circle shift = +14°). Note that the outer circle is always shifted by +12°.

In the second experiment, we tested the hypothesis that a planned, but not executed, movement in a specific direction could bias participants’ perception. Indeed, motor planning can have the same perceived consequences as actual movements (Garbarini et al., 2012; Piedimonte et al., 2015) and can influence perception (Wohlschläger, 2000). We tested the hypothesis that the intention to move could also interfere with or facilitate exogenous directional cues. Thus, we replaced the execution of movement from Experiment 1 with the intention to do so, asking participants to not execute the hand movements but to plan them (see **Figure 5**).

The first (No-Planning) condition was identical to the No-movement condition of Experiment 1. In this condition we predicted the same results as in the first experiment, that the inner circle rotation would bias participants’ perception of the outer circle rotation.

In the second (Planning) condition, participants received two instructions (duration = 1000 ms): green brackets ([]) indicating a rightward (CW) hand rotation or red brackets indicating a leftward (CCW) hand rotation. After the brackets disappeared, after 90 ms the circles appeared and began rotating. Subjects were asked to report the direction of the outer circle’s rotation. After the researcher recorded the judgment, the circles disappeared and participants approached the touch-screen with their right hand and performed the cued movement. After the movement was performed the screen turned black for 1000 ms before the beginning of the next trial. This condition was designed such that participants were required to plan the movement while judging the outer circle rotation (i.e., the time between the instruction and the black screen where they have to perform the movement). In this last condition our predictions were that: if the intention to move was the critical ingredient of the executed movement effects, we would observe the same results as in the first experiment. However, if the implementation of the motor act was the critical ingredient, then intended hand movements would not have the same effect as executed hand movements.

The same number of randomized trials per participant (240) as in the first experiment was used and conditions were counterbalanced within subjects in this order: No-Planning, Planning, Planning, No-Planning. The experimenter present in the room registered verbal reports and made sure that all participants performed the correct movements required in the Planning condition. If a participant performed a wrong movement during a trial, the trial was repeated.

### Analysis and Results

**FIGURE 6:**
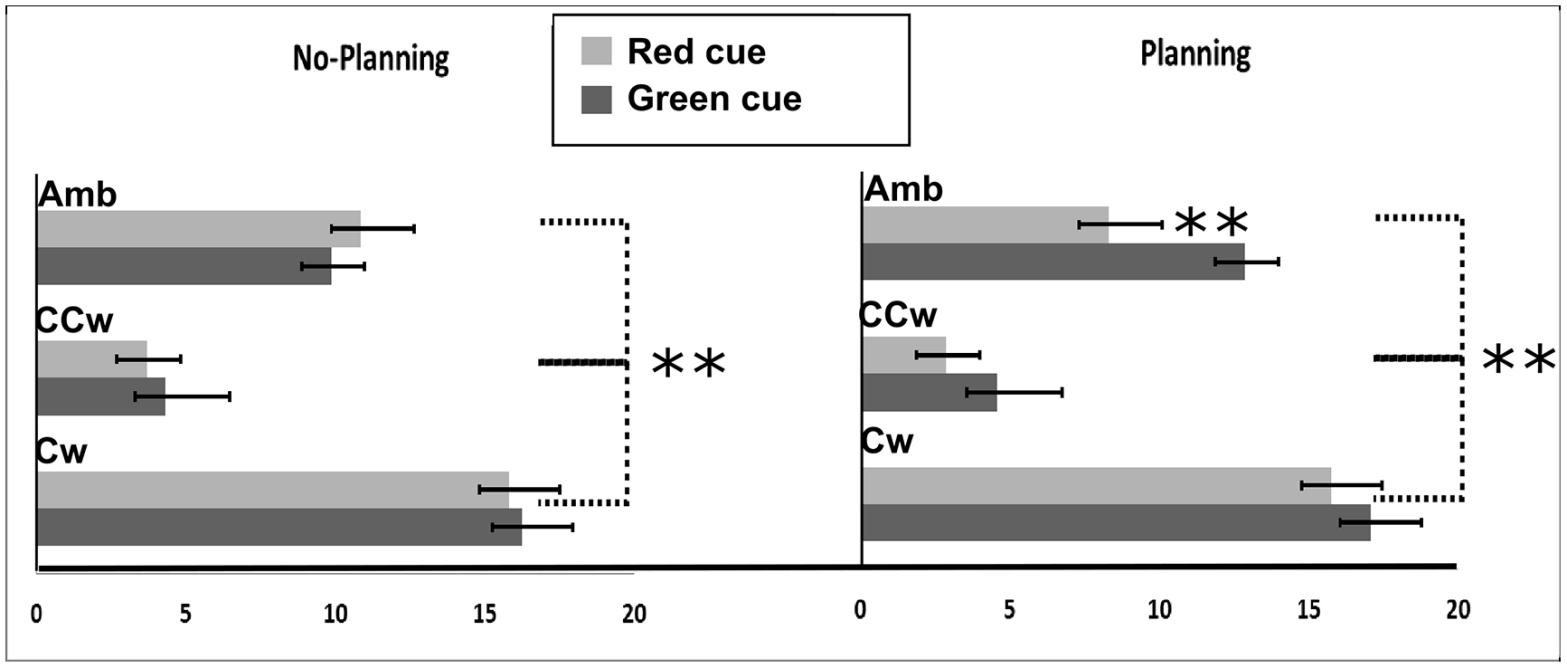
**Experiment 2 results.** On the *X*-axis, PI is showed (range: 0, +20). 0 represent a totally CCW biased perception while +20 represent a totally CW biased perception. On the *Y*-axis Inner circle rotations are described: Amb = ambiguous inner circle rotation; CCw = counterclockwise circle rotation; Cw = clockwise inner circle rotation. Error bars represent SEM. Light gray bars represent red cues and dark gray bars represent green cues. Asterisks represent significant results: ^∗∗^*P* < 0.01.

All main results are illustrated in **Figure 6**. In particular, on the *Y*-axis, inner circle rotations are described (Amb = ambiguous inner circle rotation, +12°; CCw = counterclockwise inner circle rotation, -14°; Cw = clockwise inner circle rotation, +14°) while on the *X*-axis the value of PI is showed (between 0 and 20). Light gray bars represent red cues and dark gray bars represent green cues while error bars represent SEM. Asterisks represent significant results (^∗∗^*P* < 0.01). On the left part of the **Figure 6**, results from the No-Planning condition are showed while in the right part of the **Figure 6** results from the Planning condition are showed.

Using PI as the dependent variable, we performed a 2 × 3 × 2 repeated measure ANOVA with three main factors: Condition (two levels: No-Planning or Planning), Inner rotation (three levels: AMB, CCW, CW), and Cue (two levels: red, green). The analysis showed significant main effects of Inner Rotation [*F*(2,30) = 190.08; *P* < 0.001, $\eta_{p}^{2}$ = 0.92], Cue [*F*(1,15) = 11.33; *P* = 0.004, $\eta_{p}^{2}$ = 0.43] and significant interactions between Condition and Cue [*F*(1,15) = 19.45; *P* = 0.00^∗^, $\eta_{p}^{2}$ = 0.55] and Condition, Inner Rotation, and Cue [*F*(2,30) = 7.59; *P* = 0.002, $\eta_{p}^{2}$ = 0.33]. Newman–Keuls *post hoc* test on Inner Rotation significance confirmed a general difference between all three rotations: AMB, CCW and CW (for each comparison: *P* < 0.001). As in the first experiment, in both conditions (No-Planning and Planning), when the inner circle was perceivable as rotating ambiguously participants did not have a specific bias in judging the outer circle rotation (mean = 10.48). When the inner circle was perceivable as rotating CCW, participant had a bias toward perceiving the outer circle as rotating CCW more often (mean = 3.84). Finally, when the inner circle was perceivable as rotating CW, participant had a bias toward perceiving the outer circle as rotating CW more often (mean = 16.22).

*Post hoc* analysis of the interaction between Condition, Inner Rotation, and Cue showed, in the No-Planning condition, no significant difference between the green and the red cue meaning that participants were only influenced by the inner circle rotations in judging the outer circle rotation. In the Planning condition we found a significant difference in the perceived rotations between red and green cues when the inner circle was rotating ambiguously (Newman–Keuls *post hoc* test, *P* < 0.001). In this case planned actions influenced their perception of the AMB movement of the outer circle. Participants perceived significantly more CCW rotations when a red cue was displayed (mean = 8.315) and more CW rotations when a green cue was displayed (mean = 12.875). However, when the inner circle was rotating CCW or CW, the direction of the planned movement did not make a difference (**Figure 6**).

Planned comparisons were used to check *a priori* predictions about interference or facilitation effects between planned hand movements and inner circle rotations. As shown in the *post hoc* analysis, planned movements did not show facilitation or interference effects between hand movements and inner circle rotations.

### Discussion

Results from Experiment 2 confirmed that the inner circle rotation, when not AMB, biased participants’ perception of the outer circle AMB rotation. Also, when participants planned to move their hand in a specific direction and both circles were perceivable as ambiguously rotating, participants’ perception was biased in the same direction of the movement they planned. This result is in line with studies on the programming components of movements that show that these planned, yet not executed, actions have the same effects as actual movement (Wohlschläger, 2000; Garbarini et al., 2012).

When planned movements and inner circle rotations were in opposite directions, only the inner circle rotations biased participants’ perception. This result mimicked the results of the first experiment. Finally, when planned hands rotation and inner circle rotations had the same direction we did not find the same facilitation effect found in the first experiment.

## Experiment 3

### Procedure and Conditions

**FIGURE 7:**
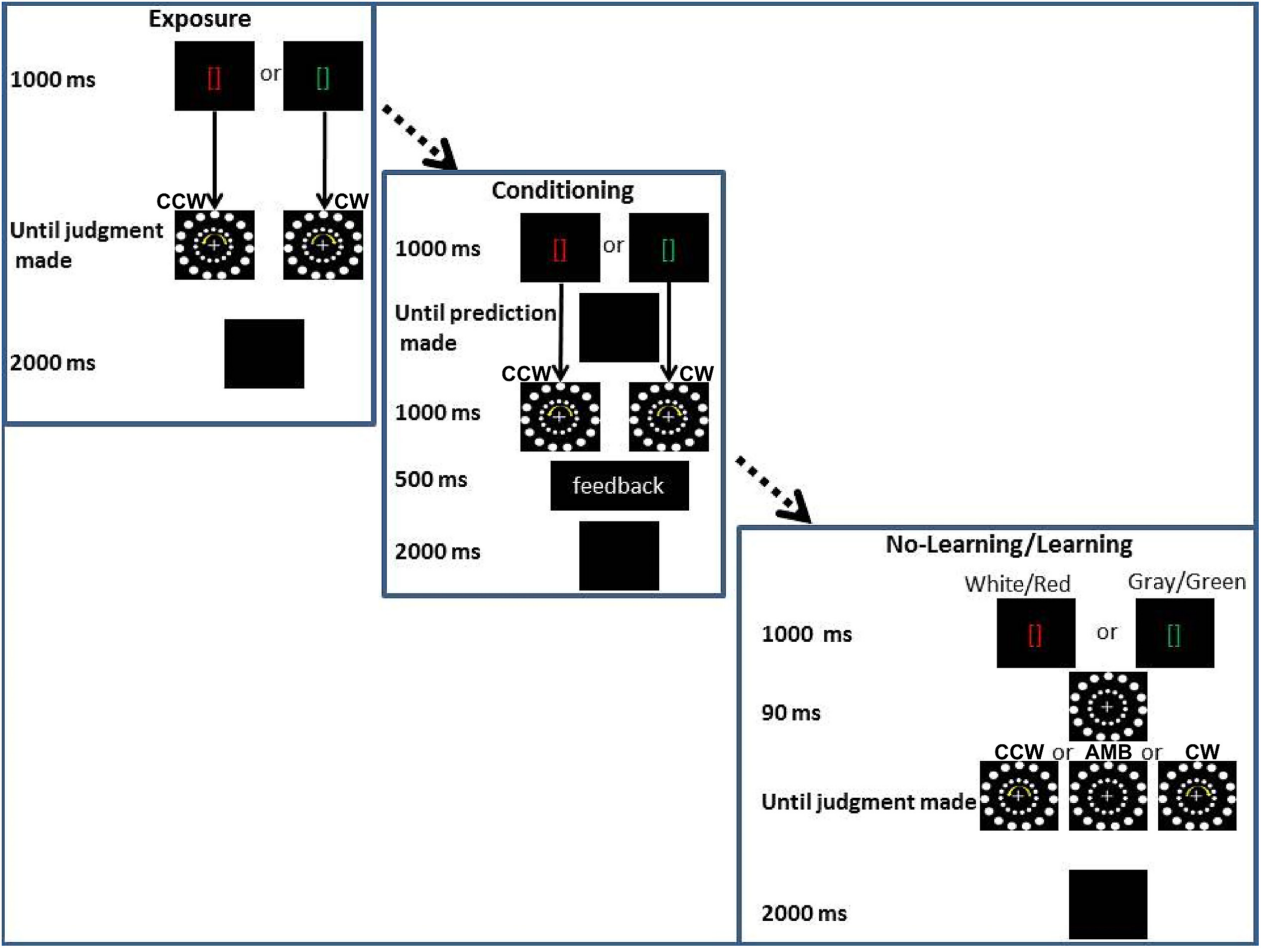
**Experiment 3 conditions.** From the upper corner to the lower right corner: Exposure training, (Association training) and experimental conditions (No-Association and Association). In the bottom right corner, the two main experimental conditions are shown together, the only difference being the color of the brackets: gray and white for the No-Association condition and red and green for the Association condition. CCW = counterclockwise (inner circle shift = -14°), AMB = Ambiguous (inner circle shift = +12°), CW = clockwise (inner circle shift = +14°). Note that the outer circle is always shifted by +12°.

In the third experiment, we tested the hypothesis that learned knowledge about the AMB motion could bias participants’ perception while another source of biasing information (i.e., another circular motion) was also displayed. Such knowledge would be another form of endogenous information, a form not tied to the motor systems. All participants went through three stages of training before completing the two experimental conditions (see **Figure 7**).

The first part of training was similar to Experiments 1 and 2 (see Stimuli and Apparatus common to all experiments). In the second part of training, Exposure Training, red or a green brackets were displayed (duration = 1000 ms). After green brackets, only a CW stimulus was shown (i.e., the inner circle was shifted by 14° in each frame) while after the red brackets, only a CCW stimulus was shown (i.e., the inner circle was shifted by -14° in each frame). AMB stimuli (i.e., with the inner circle shifted by 12° in each frame) were not shown. In the Exposure Training, as in all the experiments, participants judged the rotation of the outer circle. After their response, the screen went black for 2000 ms and another trial started. The Exposure Training was used to allow the participants to learn the association (“after a green bracket, a CW rotation will be displayed” and “after a red bracket, a CCW rotation will be displayed”). Twenty trials for each association (green-CW, red-CCW) were used.

In the last part of the training, Association Training, a red or a green bracket was displayed (duration = 1000 ms) and then the screen turned black. During this period, participants had to “predict” the direction of rotation that followed the brackets. After the prediction was made, a CW stimulus was shown following the green brackets and a CCW stimulus was shown following the red brackets (duration = 1000 ms). Accuracy feedback was given to the participants in the middle of the screen for 500 ms. “Correct” was shown if the participants answered “CW” after the green brackets or if they answered “CCW” after the red brackets. “Incorrect” was shown in the other two opposite cases. This last training session was designed to strengthen their association between the colored cues and the rotation of stimuli. Ten trials for each association (green-CW, red-CCW) were used (total *n* trials = 20).

After Association Training, each participant underwent two conditions. The first (No-Association) condition mimicked the No-Movement and No-Planning conditions of the previous experiments with the only difference that the brackets in the middle of the screen were white or gray. In this condition we expected to observe the same results as in the first and second experiments. In this case, participants’ reports would be captured by the inner circle rotations and they would judge the outer circle direction as rotating in the same direction as the inner one. When both circles were perceivable as ambiguously rotating we predicted that participants would tend to have the same frequency of CW or CCW perceptions. These results would replicate results of the first condition of Experiments 1 and 2 and would confirm that the inner circle movement can bias participants’ perception.

In the second (Association) condition, the only difference with the previous condition was that the white or gray brackets were substituted with green or red brackets (the same color as in the training). In this condition we predicted that these cues would interact with the inner circle rotation. If the inner circle was perceivable as ambiguously rotating, we expected that participants would perceive more CW rotations when seeing a green cue and more CCW rotations when seeing a red cue. If the inner circle was clearly perceivable as rotating in a certain direction (CW or CCW), we expected participants to be less captured by the inner circle rotation when the cue represented the opposite direction (interference) and to be more captured by the inner circle if the cue was indicating the same direction as the inner circle rotation (facilitation). These results would demonstrate that learned knowledge can biasing participants’ perception. Alternatively, if previously learned information has no effect on motion perception, we expected participants to be biased only by the rotation of the inner circle (i.e., exogenous information).

As in the first two experiments, 240 trials per participant were recorded and conditions were counterbalanced within participants in this order: No-Association, Association, Association, No-Association.

### Analysis and Results

**FIGURE 8:**
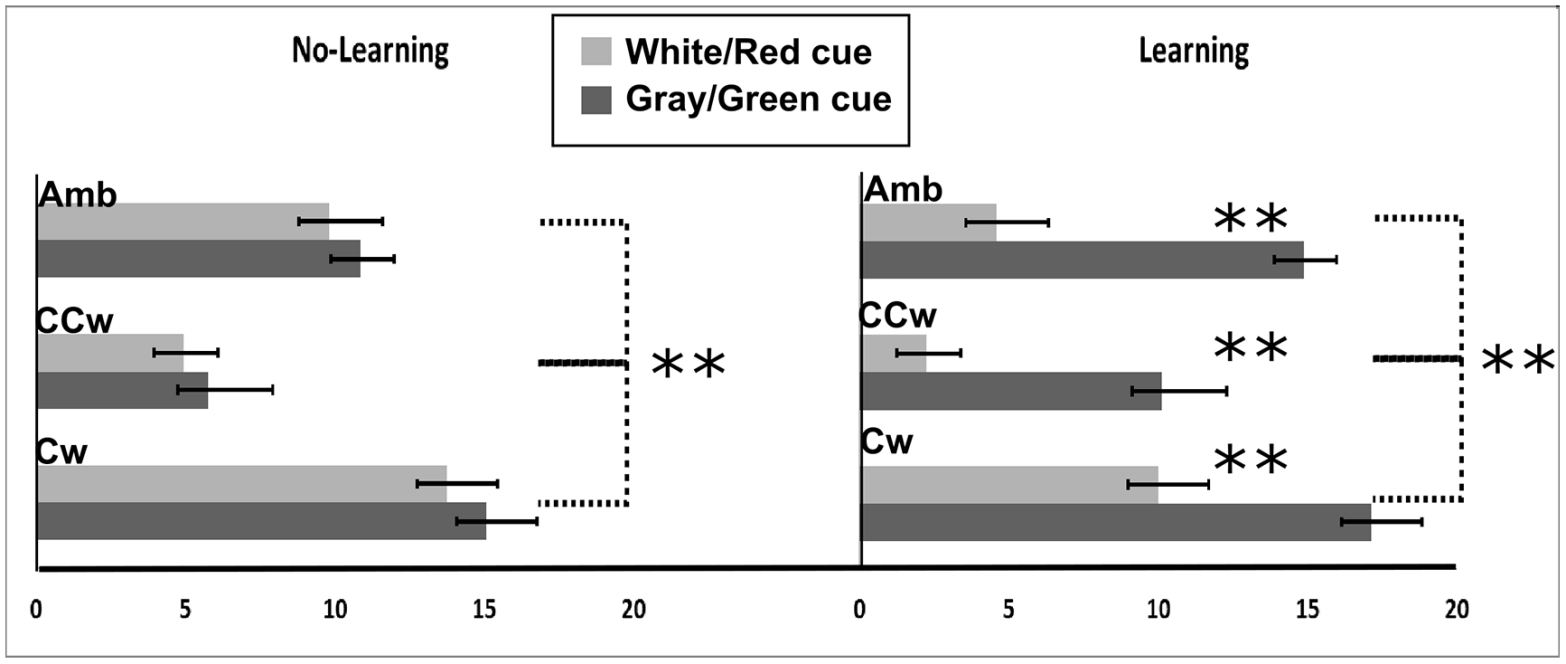
**Experiment 3 results.** On the *X*-axis, PI is showed (range: 0, +20). 0 represents a totally CCW biased perception while +20 represents a totally CW biased perception. On the *Y*-axis Inner circle rotations are described: Amb = ambiguous inner circle rotation; CCw = counterclockwise circle rotation; Cw = clockwise inner circle rotation. Error bars represent SEM. Light gray bars represent white (in the No-Association condition) or red (in the Association condition) cues and dark gray bars represent gray/green cues. Asterisks represent significant results: ^∗∗^*P* < 0.01.

All main results are illustrated in **Figure 8**. In particular, on the *Y*-axis, inner circle rotations are described (Amb = ambiguous inner circle rotation, +12°; CCw = counterclockwise inner circle rotation, -14°; Cw = clockwise inner circle rotation, +14°) while on the *X*-axis the value of PI is showed (between 0 and 20). Light gray bars represent red cues and dark gray bars represent green cues while error bars represent SEM. Asterisks represent significant results (^∗∗^*P* < 0.01). On the left part of the **Figure 8**, results from the No-Association condition are showed while in the right part of the **Figure 8** results from the Association condition are showed.

The PI was the dependent variable in a 2 × 3 × 2 repeated measure ANOVA with three main factors: Condition (two levels, No-Association or Association), Inner rotation (three levels: AMB, CCW, CW), and Cue (two levels: red/white, green/gray). The analysis showed significant main effects of Inner Rotation [*F*(2,30) = 94.44; *P* < 0.001, $\eta_{p}^{2}$ = 0.86], Cue [*F*(1,15) = 54.49; *P* < 0.001, $\eta_{p}^{2}$ = 0.78], and significant interactions between Condition and Cue [*F*(1,15) = 22.81; *P* < 0.001, $\eta_{p}^{2}$ = 0.60] and Condition, Inner Rotation, and Cue [*F*(2,30) = 4.06; *P* = 0.02, $\eta_{p}^{2}$ = 0.21]. Newman–Keuls *post hoc* test on Inner Rotation significance confirmed a general difference between all three rotations: AMB, CCW, and CW (for each comparison: *P* < 0.001). In both conditions (No-Association and Association), when the inner circle was perceivable as rotating ambiguously participants did not have a specific bias in judging the outer circle rotation (mean = 10.03). When the inner circle was perceivable as rotating CCW, participant had a bias toward perceiving the outer circle as rotating CCW (mean = 5.76). Finally, when the inner circle was perceivable as rotating CW, participant had a bias toward perceiving the outer circle as rotating CW (mean = 13.94).

*Post hoc* analysis showed no significant difference between the white and the gray cue. Thus, in this condition, participants were only biased by the inner circle rotations in judging the outer circle rotation. In the Association condition, using Newman–Keuls *post hoc* analysis, we found a significant difference in the perceived rotations between red and green cues when the inner circle was rotating ambiguously, CCW or CW (for each comparison, *P* < 0.001). When the inner circle rotated ambiguously, participants perceived significantly more CCW rotations when a red cue was displayed (mean = 4.56) and more CW rotations when a green cue was displayed (mean = 14.87). When the inner circle was rotating CCW, participants perceived significantly more CCW rotations when a red cue was displayed (mean = 2.25) and did not show any bias when a green cue (i.e., linked to an opposite CW direction) was displayed (mean = 10.25). When the inner circle was rotating CW, participants perceived significantly more CW rotations when a green cue was displayed and did not show any bias when a red cue (i.e., linked to an opposite CCW direction) was displayed (mean = 10).

Finally, we conducted planned comparisons to check *a priori* predictions about interference or facilitation effects of previously learned knowledge. Specifically, participants reported significantly more CCW outer circle rotations when they saw red cues (related to a CCW direction) and the inner circle was perceivable as rotating CCW with respect to when they only saw the inner circle rotating CCW with a cue not related to any learned knowledge about the rotation [*F*(1,15) = 9.05; *P* = 0.008]. When participants saw green cue (related to a CW direction) and the inner circle was perceivable as rotating CW, they reported significantly more CW outer circle rotations compared to when they only saw the inner circle rotating CCW with a cue not related to any learned knowledge about the rotation [*F*(1,15) = 12.61; *P* = 0.003]. Planned comparison also showed an inhibitory effect. When participants received a green cue (CW) and the inner circle was perceivable as rotating CCW, they reported significantly more CW outer circle rotations as compared to when they only saw the inner circle rotating CCW and they didn’t receive a cue related to any learned knowledge about the rotation [*F*(1,15) = 10.16; *P* = 0.006]. Also, when participants received a red cue (CCW) and the inner circle was perceivable as rotating CW, they saw significantly more CCW outer circle rotations as compared to when they only saw the inner circle rotating CCW and they didn’t receive a cue related to any learned knowledge about the rotation [*F*(1,15) = 10.51; *P* = 0.006], (**Figure 8**).

### Discussion

In the last experiment participants learned an association about the inner circle motion. Specifically, after a colored cue participants expected a certain inner circle rotation (red-CCW, green-CW). While participants learned this associative rule during the training, in the experimental conditions the inner circle rotated CCW, CW, or ambiguously without a direct link to the colored cues.

When gray and white cues were shown, the inner circle rotation biased participants’ motion perception confirming that in an AMB situation, an exogenous source of movement is a valuable source of information. This result replicated the findings of Experiments 1 and 2.

Interestingly, we found that learned association of the cue to rotation of the inner circle had a greater effect on participants’ perception. When participants saw a colored cue, they reported a significantly higher number of directions linked with that specific cue in respect to when the cue was not informative. This first result is in line with previous results on associative learning and AMB motion perception (Haijiang et al., 2006). Also, this effect significantly interfered or facilitated the biasing effects of the inner circle rotation. This last result shows that a learned association can influence motion detection in AMB contexts and can even mitigate an opposite exogenous motion cue.

## General Discussion

From an evolutionary point of view, perceiving the direction of movement in the environment is a critical functions of our brain (Albright and Stoner, 1995). However, in dynamic and sometimes AMB environments, it can be difficult to determine the direction of movement of specific objects of interest like for instance when we are driving our car on a rainy day and simultaneously trying to understand where other cars are going. We used apparent motion displays with multistable stimuli, in which movement direction was AMB (Leopold and Logothetis, 1999) to study how human perception can be biased.

Many studies show that cues from the environment influence how we see the world (exogenous cues) in a bottom–up fashion. From a top–down perspective, instead, it has been showed that our movements and the planning of movements also bias our perception (endogenous cues). The present study aimed to better understand how we collate different sources of information to determine whether an object is moving in a certain direction.

From a general point of view, our experiments confirmed that perception of an AMB motion stimulus is composed by both kind of processes (top–down and bottom–up) but the interaction between the two seems to biased in favor of the bottom–up information.

Indeed, we found that participants naturally grouped the outer circle rotation (i.e., the AMB motion to be judged) with the inner circle rotation (i.e., the exogenous source of information). This grouping effect influences our perception of the direction of the AMB motion. When information from the environment is AMB, our movements and even plans to move also bias our perception of motion. In particular, if an exogenous motion is intrinsically AMB and we are moving or planning to move in a specific direction, our motion perception is biased by direction of our executed or planned movements. These results are in line with a classic premotor theory of attention where visual attention is directly linked to motor systems (Rizzolatti and Craighero, 1998) and studies on intentional neglect. Human behavioral and imaging studies demonstrate that the attentional selection of stimuli is affected by our plans to act. Approaching a cup to drink requires selecting the handle to grasp it, while approaching the same object to wash it does not require focus on the same part of the cup (Iacoboni et al., 2005). According to the “premotor theory of attention,” spatial attention is not conceived as a dedicated control mechanism, but as a weaker activation of the same frontal–parietal circuits that determine motor behavior toward specific locations (Rizzolatti et al., 1987; Rizzolatti and Craighero, 1998). Another example of motor influence on perception can be found in discussions of intentional neglect. Unilateral spatial neglect is a syndrome in which patients are unaware of, or fail to act in or toward space on the opposite side of their brain lesion. Different studies have tried to dissociate where patients are directing their attention from where they are moving their limbs, i.e., the intentional component (Chatterjee and Coslett, 2003). For instance: in a neglect patient it is has been observed that using limbs to point, instead of verbally reporting the presence of a target improved the task performance, indicating that motor responses can modulate sensory discriminability (Ricci and Chatterjee, 2004). Furthermore, studies using fMRI and stimulation techniques demonstrate specific functional links between the activation of brain areas related to apparent motion perception, like area MT, and the activation of areas related to motor execution, like primary motor cortex (Antal et al., 2004) and motor planning like supplementary motor area (Schubotz and von Cramon, 2001). These networks could represent the neural substrate for the effects observed in our experiments with executed or just the planning of movements. Since a bias in motion perception occurred with planned, yet not executed, movements, primary motor cortex might not be a necessary part of the network involved in creating these biases.

Performing or planning movements in an opposite direction compared to the exogenous cue did not modulate the perceptual bias driven by the external source of information. Ecologically speaking, this relative importance of exogenous cues might have a greater evolutionary value. Tracking movements of prey (e.g., a bird in the midst of its flock) or checking movements of dangerous animals might be of primary importance and thus relatively resistant to modulation (Thompson, 2003). Thus, from the first and the second experiment we found that bottom up information is very “resistant” to the top–down streaming of information arising from our motor programming system, at least with this kind of stimuli. However, it has to be noted that that the kind of bottom up feature that participants could extract from our stimuli was a spatiotemporal figural overlap (i.e., both the outer AMB circle and the inner, sometimes less AMB, circle rotated with the same frequency and had the same disposition and dots number) is considered one of the strongest form of grouping, that is one of the strongest visual bottom–up or stimulus driven factor (Tse and Cavanagh, 2000). Thus, it is possible that other classical form of bottom–up features in apparent motion perception such as spot proximity (Ullman, 1979) could be less “resistant” to top–down influences from motor execution and planning.

Still, one kind of endogenous information seems influential in discerning movements: past experience. Movement execution and intention to move represent part of our procedural knowledge. We plan and execute movements since birth, but during our development we also acquire explicit knowledge about people and moving objects (Parrish et al., 2005). Psychological states derived from past experience can influence visual perception (Bruner and Goodman, 1947; Balcetis and Dunning, 2006; Dunning and Balcetis, 2013). In our last experiment, in line with previous studies (Durgin, 2002; Haijiang et al., 2006), we found, first, that previously learned knowledge about the displayed motion completely biased participants perception of the unstable outer circle motion. Furthermore, this previous knowledge eliminated the effects of an exogenous cue in biasing participants’ perception. Thus, this kind of explicit endogenous information was more powerful than movements or motor plans in biasing participants’ perception. When the instruction (i.e., endogenous cue) was linked to an opposite inner circle direction, the otherwise robust bias of the inner circle’s actual rotation (i.e., exogenous cue) was eliminated. This result indicates that participants’ judgment of the outer circle’s AMB rotation based on their previously learned knowledge, actually modified their perception of the opposite and less AMB exogenous cue. Again, if we frame this result in an evolutionary point of view, it is plausible that an important source of information about AMB prey or predator movements was our previous associations with their specific actions. This source of information is indeed one of the strongest top–down factors in biasing motion perception and it has been showed that if this kind of knowledge about motion is present from birth it can strongly bias apparent motion perception. For instance, if a series of strokes composing a Chinese character appear all at once on a screen, participants raised in China tend to perceive the strokes in the direction they would have taken when drawn by hand while participants raised in America tend to perceive the opposite direction (Tse and Cavanagh, 2000). In our third experiment we showed that even a simple learning procedure (only 40 trials of conditioning) can represent a form of top–down source of information capable to significantly override a strong bottom–up characteristic such as grouping by spatiotemporal overlapping (i.e., the grouping occurred between the outer AMB circle and less AMB inner circle).

However, it is possible that effects observed in the experiments maybe linked to the specific inner circle rotation, that is the specific degrees of rotation applied to this exogenous cue. Further experiments should focus on different degrees of rotation (i.e., different “strength” levels) of the exogenous cue. Also, another intrinsic problem in studying bistable stimuli and their interaction with kinesthetic information is that it is difficult to differentiate between attentional “only” effects and effects derived directly from the motor system. We partially addressed this issue using a small lag between when participants performed hand movements and the start of circles’ rotation, thus adding a temporal congruency constraint known to be crucial in multi-sensory integration studies (Soto-Faraco et al., 2003; Hu and Knill, 2010). To fully solve this problem, further experiments should apply this paradigm to stable stimuli like one-dimensional grating patterns behind a circular aperture (Hu and Knill, 2010). Finally, it has to be considered that participants, at least in the No-Planning condition, could have used different cognitive strategies to disambiguate the outer circle rotation. That is, for instance, participants could have linked the red cue to a CCW rotation or the green cue to a CW rotation as in the third experiment, without really focusing on planning any movement. A way to reduce the chance of this problem arising in future studies would be to mix planning and no-planning trials in the same run so that, if a cognitive strategy is present (e.g., linking red cues to CCW), this strategy should be present in both planning and no-planning trials (since they both have red and green cues) and thus should be more detectable and less hidden by the experimental design (i.e., the No-Planning condition separated from the Planning condition) used in this study.

## Conclusion

Our study showed that we can use different sources of information to disambiguate AMB motion. We can rely on exogenous information to predict movements of AMB targets. Also, our own movements and even plans to move can bias our perception of AMB motion. However, in our study, movement execution seem to only facilitate biases produced by exogenous cues (but do not seem to counter them) while movement planning seems to have a lesser effect, since it neither facilitates nor counters biases produced by exogenous cues. Finally, learning also has an important effect on our perception of AMB motion. In this case, learning can counteract the effects of low-level exogenous grouping. Further studies, with more ecological experiments (e.g., Nardo et al., 2011) will help us to better understand the hierarchical relationships between these different source of information that modulate our motion perception.

## Conflict of Interest Statement

The authors declare that the research was conducted in the absence of any commercial or financial relationships that could be construed as a potential conflict of interest.
